# 

**Published:** 1883-07

**Authors:** 


### SOUTHERN DENTAL ASSOCIATION.

The Fifteenth Annual Session of the Southern Dental Association will meet in Atlanta, Georgia, Fifth Tuesday in July, (31st), 1883.

*Officers.*—L. D. Carpenter, President, Atlanta, Ga.; J. M. Riggs, First Vice-President, Hartford, Conn.; R. A. Holliday, Second Vice-President, Atlanta, Ga.; J. R. Walker, Third Vice-President, New Orleans, La.; H. J. McKellops, Fourth Vice-President, St. Louis, Mo.; J. F. Thompson Fifth Vice-President, Fredericksburg, Va.; J. P. Holmes, Corresponding Secretary, Macon, Ga.; W. H. Hoffman, Recording Secretary, Charlotte, N. C.; H. A. Lawrance, Treasurer, Athens, Ga.

*Executive Committee.*—J. H. Coyle, Thomasville, Ga.; T. M. Allen, Eufaula, Ala.; J. B. Patrick, Charleston, S. C.; R. A. Holliday, Atlanta, Ga.; A. G. Bouton, Savannah, Ga.

STANDING COMMITTEES.—*Dental Education.*—J. P. H. Brown, *Chairman*, Augusta, Ga.; A. C. Ford, Fernandina, Fla.; W. H. Marshall, Oxford, Miss.; Charles L. Steel, Richmond, Va.

*Dental Hygiene.*—R. F. Hunt, *Chairman*, Washington, City, D. C.; G. B. White, Chester, S. C.; E. S. Chisholm, Tuscaloosa, Ala.; R. A. Freeman, Nashville, Tenn.; S. G. Holland, Atlanta, Ga.

*Pathology and Therapeutics.*—J. M. Riggs, *Chairman*, Hartford, Conn.; J. B. Patrick, Charleston, S. C.: G. F. S. Wright, Columbia, S. C.; G. F. Fredrick, New Orleans, La.; E. L. Hunter, Enfield, N. C.

*Histology and Microscopy.*—Wm. H. Atkinson, *Chairman*, New York City; W. W. H. Thackston, Farmville, Va.; H. J. McKellops, St. Louis, Mo.

*Chemistry.*—J. R. Walker, *Chairman*, New Orleans, La.; H. J. Royal, Savannah, Ga.; W. S. Dwinelle, New York City; D. Hopps, Savannah, Ga.

*Operative Dentistry.*—J. H. Harris, *Chairman*, Baltimore, Md.; A. G. Bouton Savannah, Ga.; S. B. Barfield, Macon,

Ga.; Theo. Chupein, Philadelphia, Pa.; W. S. Brown, Charleston, S. C.; T. M. Allen, Eufaula, Ala.

*Mechaincal Dentistry.*—J. B. Hodgkins, *Chairman*, Baltimore, Md.; H. H. Keith, St. Louis, Mo.; M. S. Jobson, Perry, Ga.; W. W. Evans, Washington City, D. C.; L. M. Cowardin Richmond, Va.; J. C. Uhler, Baltimore, Md.

*Dental Literature.*—F. J. S. Gorgas, *Chairman*, Baltimore, Md.; A. O. Rawls, Lexington, Ky.; W. C. Wardlaw, Augusta Ga.; G. W. H. Whitaker, Sandersville, Ga.; A. F. McLaine, San Rafael, Cal.

*Volunteer Essay.*—N. W. Kingsley, *Chairman*, New York City; E. E. Hamner, Galveston, Texas.; D. E. Everitt, Raleigh, N. C.; T. C. Moore, Columbia, S. C.; I. T. Calvert, Spartanburg, S. C.; J. L. Wilborn, Grenada, Miss.; S. H. Henkle, Charlotte, Va.; J. G. McAuley, Selma, Ala.

*Appliance and Improvements.*—W. G. A. Bonwill, *Chairman*, Philadelphia, Pa.; W. W. Ford, Macon, Ga.; M. W. Foster, Baltimore, Md.; S. A. White, Savannah, Ga.; M. A. Bland, Charlotte, N. C.

*Arrangements.*—Sam'l Hape, *Chairman*, Atlanta, Ga.; S. G. Holland, Atlanta, Ga.; R. A. Holliday, Atlanta, Ga.

*Clinics.*—E. Parmley Brown, *Chairman*, Flushing, L. I.; W. G. A. Bonwill, Philadelphia, Pa.; J. W. Hunter.

*Place of Meeting.*—The United States Court Room, (Post Office Building).

*Excursion to Tallulah Falls.*—The Committes of arrangements will give an Excursion to the beautiful Falls of Tallulah, where an old-fashinded Southern Barbecue will be served. All Dentists, their wives and daughters are cordially invited.

Board can be had at the Kimball House at from $2.50 to $4.00 per day, and other places, at from $1.00 up.

SECTION 11. "It shall be the duty of each of the members of the different Committees to make an individual report, so far as such a report can be made, and in case of inability to be present at the meeting where that report is due, to forward it to the Recording Secretary, from whom it can be obtained by the Chairman of each Committee, respectively, at the time of the assembling of the Association."

By Order of the President:

L. D. CARPENTER,
*Atlanta, Ga.*

W. H. HOFFMAN,
RECORDING SECRETARY,
*Charlotte, N. C.*

J. P. HOLMES,
CORRESPONDING SECRETARY,
*Macon, Ga.*

---

### PITTSBURGH DENTAL ASSOCIATION.

The Ninth Annual Meeting of the Pittsburgh Dental Association was held the 1st Tuesday of May, 1883. The following Officers and Delegates were elected.

*President,* Dr. F. A. Reinhart; *Vice-President,* Dr. H. L. Reinecke; *Secretary,* Dr. W. H. Fundenberg; *Treasurer,* Dr. W. A. Lee. *Censors.*—Drs. W. F. Fundenberg, Williams and Goshorn.

*Delegates to American Association.*—Drs. Deitering, Diehl, Reinecke, Reinhart and W. F. Fundenberg.

*Delegates to Pennsylvania State Society.*—Drs. Goshorn, Troth, England, Williams and Nayloy.

W. H. Fundenberg, *Secretary.*
330 PENN AVE, *Pittsburgh, Pa.*

### To Readers and Correspondents.

Communications intended for publication, and exchange journals and papers, should be addressed to the Editor of the American Journal of Dental Science, 259 N. Eutaw St., Baltimore, Md. All letters relating to the business of the journal, or containing cash remittances, to be directed to Messrs. Snowden & Cowman. Articles for publication should be received by the editor at least three weeks previous to the first of the month on which the number in which it is designed for them to appear, is issued.

### MONTHLY SUMMARY.

Inebriety of the Teeth....141
To Stop Hiccough....143
To Disguise the Oder of Iodoform....143
How to Keep Cool....144
Gluten....144

☞ The American Journal of Dental Science will contain fifty pages of reading matter in each number, making a volume of about

*Six Hundred* pages,

EXCLUSIVE OF ADVERTISEMENTS.

THE

### AMERICAN JOURNAL

OF

### DENTAL SCIENCE.

The *Journal* is issued on the first of each month and contains not less than *fifty* pages of reading matter in each number, exclusive of advertisements, making a volume of *six hundred* pages per annum.

In each number will be found Original Communications, Correspondence, Selected Articles, Monthly Summary, Bibliographical Notices and Editorial Matter, and every effort will be exerted to render the *Journal* worthy of the patronage of the Dental profession.

A generous co-operation is respectfully solicited from the members of the profession ; and as the *Journal* has now a printing office of its own, which materially lessens the cost of its publication, it will be issued at the reduced subscription price of

**$2.50 Per Annum in Advance.**

Communications are respectfully solicited on all subjects pertaining to practice of dentistry, as well as reports of casses occurring in dental practice.

### RATES OF ADVERTISING.

| | 1 MO. | 2 MO. | 3 MO. | 6 MO. | 12 MO. |
|---|---|---|---|---|---|
| 1 PAGE. | $15 00 | $22 50 | $30 00 | $50 00 | $100 00 |
| ½ " | 10 00 | 15 00 | 20 00 | 30 00 | 50 00 |
| ¼ " | 7 00 | 11 00 | 15 00 | 20 00 | 30 00 |
| ⅛ " | 5 00 | 8 00 | 11 00 | 15 00 | 20 00 |

All original communications designed for publication, works for review, new instruments for notice, exchanges, &c., should be directed to the editor, 259 N. Eutaw St,, Baltimore, Md.

All advertisements and subscriptions should be address to

SNOWDEN & COWMAN,

*Publishers of the Am., Juornal of Dental Science.*

86 W. FAYETTE ST., BET., CHARLES & LIBERTY STS.,

BALTIMORE, MD.

